# MRI-guided focused ultrasound: new hope for drug-resistant neurological conditions

**DOI:** 10.1007/s00415-023-12024-2

**Published:** 2023-10-03

**Authors:** S. Bari, N. P. Robertson

**Affiliations:** 1 Department of Neurology, University Hospital of Wales, Heath Park, Cardiff, CF14 4XN UK; 2 Institute of Psychological Medicine and Clinical Neuroscience, Cardiff University, University Hospital of Wales, Heath Park, Cardiff, CF14 4XN UK

Since the therapeutic use of focused ultrasound was first described in 1932, the technology has developed significantly, particularly over the last decade. With the advent of new imaging modalities, such as fMRI and PET, it is now possible to pinpoint specific body areas and destroy pathologic lesions with Focused Ultrasound Ablation (FUSA). FUSA has potential for treating intracranial lesions employing several focused ultrasonic beams. While individual beams are harmless, when concentrated on a small area it can result in thermal and mechanical destruction. Some effects, such as tissue destruction and clot lysis are permanent, whilst others such as blood–brain barrier disruption and neuromodulation may be transitory or reversible. It is noninvasive and requires no anaesthetic, so may have particular advantages for elderly and/or frail patients. In 2016, the FDA approved focused ultrasound for essential tremor and then tremor-dominant PD in 2018. There are now 25 categoric approvals for the use of FUSA worldwide and a large number of trials are currently addressing additional indications. In this month’s journal club, we describe three recent studies, aiming to establish the efficacy and safety of focused ultrasound techniques in Essential Tremor, Refractory Epilepsy, and Parkinson’s disease.

## Magnetic resonance imaging-guided focused ultrasound (MRgFUS) thalamotomy for essential tremor: 5-year follow-up results

The most common surgical approach to intractable disabling essential tremor (ET) remains DBS, however, not all patients are suitable. MRgFUS may be an appropriate alternative providing safety and efficacy are comparable. In this observational study, Cosgrove et al. explore long-term safety and efficacy of unilateral MRI-guided focused ultrasound (MRgFUS) thalamotomy for medication-refractory essential tremor. This study represents the longest follow-up reported for MRgFUS thalamotomy and one of the largest multi-center cohorts at 5 years for thalamotomy of any method.

Outcomes including Clinical Rating Scale for Tremor (CRST), including postural tremor scores (CRST Part A), combined hand tremor/motor scores (CRST Parts A and B), and functional disability scores (CRST Part C), were recorded by expert neurologists. The Quality of Life in Essential Tremor Questionnaire (QUEST) was used to assess quality of life. CRST and QUEST scores at 48 and 60 months post-MRgFUS were compared to those at baseline to assess treatment efficacy and durability. Forty-five and forty patients completed the 4 and 5-year follow-up respectively. CRST scores for postural tremor (Part A) for the treated hand remained significantly improved by 73.3% and 73.1% from baseline at both 48 and 60 months posttreatment (*p* < 0.0001). Combined hand tremor/motor scores (Parts A and B) also improved by 49.5% and 40.4% (*p* < 0.0001) at each time point. Functional disability scores (Part C) increased slightly over time but remained significantly improved through the 5 years (*p* < 0.0001). Similarly, QUEST scores remained significantly improved from baseline at year 4 (*p* < 0.0001) and year 5 (*p* < 0.0003). At completion of 5-year follow-up, reported adverse effects included paresthesia (8 patients), imbalance (6), unsteadiness (2), gait disturbances (2), limb weakness (2), dysmetria (2), dysgeusia (2), slow movements (1), and head pressure (1), and all were classified as mild (71%) to moderate (29%).

**Comment:** Unilateral MRgFUS thalamotomy may be an attractive treatment option for patients with refractory ET. This trial presents some compelling evidence of durability and efficacy over 5 years. No serious adverse events were reported. As a noninvasive option, unilateral MRgFUS thalamotomy for ET has every potential to replace DBS in the future.

Cosgrove GR, et al. J Neurosurg. 2022 Aug 5; 138(4):1028–1033.

## A phase one open-level trial evaluating focused ultrasound unilateral anterior thalamotomy for focal-onset epilepsy

Approximately one-third of the patients with epilepsy eventually become refractory to medication; a small number undergo surgical intervention leaving the majority with sub-optimal treatment. Previous small open-label trials have suggested efficacy of ANT (anterior nucleus of off thalamus) deep brain stimulation in treating refractory epilepsy. This pilot study describes initial safety and feasibility experience from an open-label, single-center clinical trial of ANT FUSA in treatment-refractory epilepsy. Two adult subjects with treatment-refractory, focal-onset epilepsy were recruited and received ANT FUSA using the Exablate Neuro (Insightec) system. Safety and feasibility (primary outcomes), and changes in seizure frequency (secondary outcome) were determined at 3, 6, and 12 months. Safety was assessed by the absence of side effects including new onset neurological deficits or performance deterioration on neuropsychological testing. Feasibility was defined as the ability to create a lesion within the anterior nucleus. Monthly seizure frequency was compared between baseline and post-thalamotomy. Both patients tolerated the procedure well, without neurological deficits or serious adverse events. One patient experienced a decline in verbal fluency, attention/working memory, and immediate verbal memory. Seizure frequency reduced significantly in both patients; one patient was seizure-free at 12 months, and in the second patient, the frequency reduced from 90 to 100 seizures per month to 3–6 seizures per month.

**Comment:** Seizure freedom or near seizure freedom is currently a lofty ambition in this patient population and considerable work including randomized-controlled clinical trials are still required to formally determine efficacy. Although the potential for some cognitive deficits has emerged, reported effects on seizure reduction from this very small exploratory study seem promising. Further studies are clearly required to refine indication and explore the use of ANT FUSA for refractory epilepsy.

Krishna et al. Epilepsia. 2023; 64:831–842.

## Trial of globus pallidus focused ultrasound ablation in Parkinson’s disease

Both radiofrequency pallidotomy and deep brain stimulation (DBS) using high-frequency electrical stimulation have efficacy in reducing motor symptoms in Parkinson’s disease (PD). Although DBS has become the preferred surgical option because of its ability to control bilateral symptoms and adjustability if symptoms are worsening, it remains an invasive surgical procedure compared to FUSA. FUSA involves real-time guidance with MRI to monitor tissue temperature, while ultrasound beams are gradually delivered to target tissue. A previous small open-label study demonstrated that FUSA of globus pallidus internus can improve dyskinesia and reduce the severity of motor signs on the treated side in off-medication state which is maintained for more than 1 year. This multi-center prospective double-blind randomized-controlled trial aimed to evaluate safety and efficacy of unilateral FUSA of the globus pallidus internus and was undertaken in 16 sites across, North America, Europe, and Asia.

Patients with PD with dyskinesia or motor fluctuations and motor impairment in the off-medication state were randomly assigned in a 3:1 ratio, to undergo either FUSA on the opposite side of the most symptomatic side of the body or a sham procedure. Primary outcome at 3 months was defined as a decrease of at least three points from baseline either in the score on the Movement Disorders Society-Unified Parkinson's Disease Rating Scale, part III (MDS-UPDRS III) for the treated side in off-medication state or the score on Dyskinesia Rating Scale (UDysRS) in the on-medication state. After a 3-month blinded phase, an open-label phase lasted 12 months.

Of 94 participants recruited, 69 were assigned to FUSA and 25 to the sham procedure; 65 and 22 patients completed the primary outcome assessment. 69% of participants in the FUSA group and 32% in the sham group were deemed to have responded. Of those responding in the active treatment group, 19 met MDS-UPDRS three criteria only, eight met UDysRS criteria, and 18 met both criteria. In the 3-month responders from the active treatment group, 77% continued to have a response at 12 months. Adverse events in the active treatment group include dysarthria, gait disturbance, loss of taste, visual disturbance, and facial weakness.

**Comment:** This trial suggests that unilateral pallidal ultrasound ablation resulted in a higher percentage of patients with a response in terms of motor impairment and dyskinesia scores at 3 months than sham procedure, and for most patients, improvement persisted at 12 months. However, some predictable adverse effects were reported. Limitations of the trial include missing primary outcome data for approximately 7% of patients and unsatisfactory blinding of the trial-group assignments. The lack of an expected immediate effect may be an inherent limitation of this procedure. As unilateral pallidal ultrasound ablation showed encouraging results leading to its approval by FDA and NICE, it may open the door for a trial of bilateral application of this technique.

Krishna V et al., N Engl J Med. 2023 Feb 23; 388(8):683–693.

